# Vibratory stimulation increase the electro-cutaneous sensory detection and pain thresholds in women but not in men

**DOI:** 10.1186/1472-6882-6-20

**Published:** 2006-05-23

**Authors:** Lisbeth Dahlin, Irene Lund, Thomas Lundeberg, Carl Molander

**Affiliations:** 1 Department of Public Health Sciences, division of Rehabilitation Medicine, Karolinska Institutet, SE 171 77 Stockholm, Sweden; 2 Department of Physiology and Pharmacology, Karolinska Institutet, SE-171 77 Stockholm, Sweden; 3 Rehabilitation Medicine University Clinic Stockholm, Danderyds Hospital, SE-182 88 Stockholm, Sweden; 4 Department of Rehabilitation Medicine, Uppsala University Hospital. SE-751 85 Uppsala, Sweden

## Abstract

**Background:**

Vibratory stimulation is a potential method for the treatment of pain.

**Methods:**

The effect of vibration on the forearm on detection (DT) and pain thresholds (PT) induced by electro-cutaneous stimulation were investigated in healthy male and female volunteers.

**Results:**

Women have lower baseline detection and pain thresholds as compared to men. Furthermore, women but not men report increased detection and pain thresholds after vibratory stimulation.

**Conclusion:**

Our findings indicate the potential usefulness of vibratory stimulation for pain treatment, and that gender differences should be considered in future evaluation of the method.

## Background

Vibratory stimulation is one of several non-pharmacological techniques used to reduce pain. The effects of vibration on pain has been reported in both clinical [1-8] and experimental [9-16] settings. Activation of the mechanical transient receptors is likely to be important (for review see [17]), but contribution of other more slowly adapting receptors can not be excluded [3]. Vibration activates both superficial and deeply located receptors [11,18,19]. The subsequent afferent activity in myelinated sensory axons may interact with nociceptive processing at several levels of the nervous system, including the spinal cord. One of the effects is a long lasting elevation of the pain threshold (PT) [9,14-16].

A number of experimental, clinical and epidemiological studies have shown that men and women experience pain differently; for review see ref. [20,21]. In healthy volunteers, women often report lower thresholds and tolerance to painful stimuli compared to men [21-24]. It has been suggested the gender differences are related partly to the mode of painful stimulation and pain induction method (frequency, duration, size and location) [23], the manner of presentation [20,24], but also to gender-related physiological differences [20,21,23,25,26]. In experimentally induced pain, women seem to be more sensitive to painful mechanical pressure, electrical stimulation and ischemic pain compared to men [22-24,27]. There are yet no reports on possible gender-related threshold responses to vibration stimulation.

The aim of the present study was to evaluate the effect of vibratory stimulation on electro-cutaneous detection threshold and pain threshold levels in healthy volunteers taking also the possible influence of gender into account.

## Methods

The experimental subjects were healthy student volunteers recruited from the physiotherapy programme at the Karolinska Institute. They were informed of the purpose and non-invasive experimental procedures before the experiments commenced and that they could leave at any time. The study was approved by the Ethic Committee of the Karolinska Hospital (dnr. 01-169).

### Assessment of detection and pain thresholds

The threshold assessment procedure includes non-invasive electrocutaneous stimulation of the skin of the thumb and forefinger of one hand by pressing the electrodes of the electrical stimulation unit. When reaching the respective threshold level the subjects release there fingers from the electrodes. The detection threshold (DT) was defined as the first pricking sensation and the pain threshold (PT) when the pricking sensation was altered to the first sensation of pain.

The threshold values at the respective levels were automatically recorded immediately when the fingers were released from the electrodes, but in that moment not shown to the subjects making them blind to the ongoing assessments.

The threshold assessment unit is controlled by a microprocessor (PainMatcher^®^, Cefar Medical AB, Sweden). The generated current is distributed with a monophasic rectangular pulse of 15 mA and 10 Hz. The output intensity increases by gradually widening the pulse duration in steps of 4 μs to a maximum of 396 μs, i.e. in a total of 99 steps directly related to the output. The maximum electrical charge per pulse is 5.9 μC. The contact surface area, and hence the resulting current density, is ensured by a certain load of minimum finger pressure against the electrodes. Loads between 0 and 13 kΩ secured the output of 15 mA. The numerical cut-off range is 0–99.

Both electrical DTs and PTs were recorded on four occasions separated by 10 minutes: before, during and after vibration stimulation.

### Vibratory stimulation

The vibratory stimulation (Vitamed, Germany) was applied with a rectangular probe of 13 × 20 cm to the dorsal aspect of the forearm, covering the dermatomes C5-8, with 3000 Hz, and a constant and moderate pressure for 20 minutes. The therapist was well-known to the experimental subjects.

### Statistics

The mean value and standard deviation (SD) were calculated for age. The threshold assessments were regarded as subjective estimations and the produced threshold data as ordinal data, here presented as the median and range (minimum to maximum) for the numerical units of the PainMatcher (PM) values [28,29].

The proportions of subjects with increased, unchanged, and decreased threshold values on the second occasion were calculated. The hypotheses of no change in threshold assessments between before and after vibration were analyzed by the non-parametric sign test. Mann Whitney U-test was used to analyse gender differences. A p-value less than 0.05 was regarded as significant.

## Results

Twenty-nine women (mean age 27.7, SD 6.8) and 27 men (mean age 27.7, SD 6.9) participated in this study. The assessed levels of DT and PT levels are shown in table 1.

**Table 1:** Assessed levels of detection and pain thresholds in response to vibratory stimulation to one forearm. Descriptive data, showing Pain matcher values as median value and range (min to max, numerical cut-off 0–99).

	Before		During		Immediately after		10 minutes after	
	Women, n = 29	men, n = 27	Women, n = 29	men, n = 27	women, n = 29	men, n = 27	women, n = 27	men, n = 24
Detection threshold	3 (1 to 8)	5 (1 to 9)	4 (1 to 8)	5 (2 to 8)	4 (2 to 9)	5 (1 to 9)	4 (2 to 7)	5 (2 to 8)
Pain threshold	12 (5 to 24)	15 (5 to 71)	16 (6 to 30)	17 (7 to 99)	15 (7 to 24)	18 (7 to 93)	12 (6 to 27)	19 (8 to 93)

**Figure 1:**
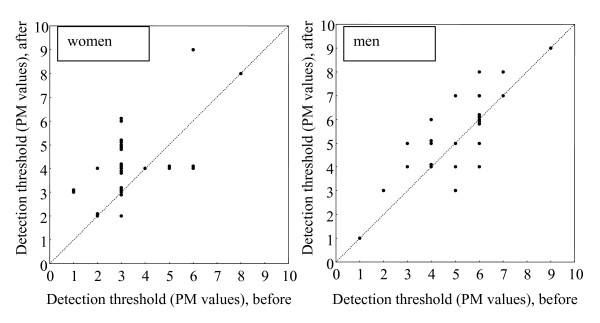
**Detection threshold**. Changes in assessed DT in women (left) and men (right). PM = pain matcher.

Immediately after the vibration, the DT were increased compared to before vibration in 16 of the 29 women (55%), unchanged in 8 (28%), and decreased in 5 (17%), p = 0.03. In men, the DT was increased in 12 of the 27 men (44%), unchanged in 10 (37%), and decreased in 5 (19%), p = 0.21, fig 1.

**Figure 2:**
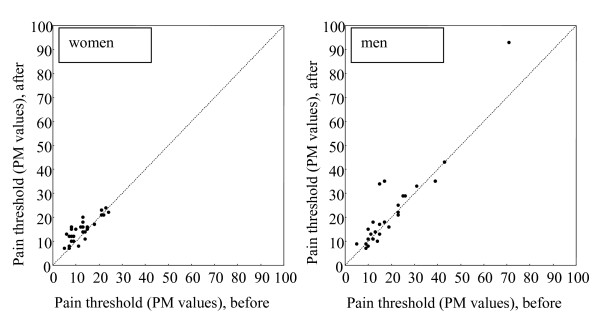
**Pain threshold**. Changes in assessed PT in women (left) and men (right). PM = pain matcher.

The PT was increased after vibration in 22 of the 29 women (76%), unchanged in 4 (14%), and decreased in 3 (10%), p = 0.005. For the men the pain threshold level was increased in 13 of the 27 men (48%), unchanged in 4 (15%) and, decreased in 10 (37%), p = 0.23, fig. 2.

**Figure 3:**
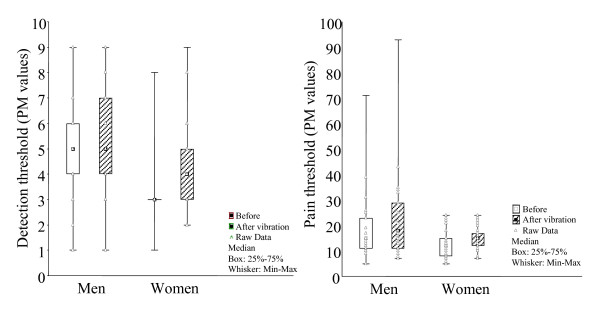
**Gender differences**. Changes related to gender in assessed DT (left) and PT (right). PM = pain matcher.

By comparing the respective threshold level in the women and men before the vibration stimulation, it was found that both the detection and pain thresholds were significantly lower in women as compared to men, p = 0.0002 and p = 0.007 respectively. After the vibratory stimulation the detection threshold levels were still lower in women than in men, p = 0.008. Despite a larger number of men than women with decreased PT after vibration, this difference was not significant (p = 0.07), fig 3.

## Discussion

### Technical considerations

Higher baseline DTs and PTs to electrical stimulation in men compared to women seems to be a frequent finding in previous studies, e.g. [22,24].

Previous studies have shown that in order to get optimal pain relief, the best site to apply the vibration probe is either in the area of pain or in dermatomes no more than two segmental levels from the pain [1,3,10,30], or in the same dermatome on the contralateral side [10], or in a trigger point outside the painful area using moderate pressure [30]. Vibration distal to the site of threshold measurement also seems to be more effective, at least during the stimulation [31]. Furthermore, findings in a previous report indicate that a larger size of the stimulus probe results in more pain relief [3]. In this study, the vibration was applied across up to four dermatomes, proximal to and in the same arm as the test stimulus, and the vibratory probe was relatively large (13 × 20 cm) with the aim to cover a reasonably large area. The 20 minutes used as standard application duration in this study is within the time interval used in previous studies, and was shown to be the shortest duration to elicit maximal pain relief for the most patients in a group with myalgia [6], but the most efficient application time for various pain states seems to be unknown. It could be that a longer application time than 20 minutes would have been more effective. Another factor which could be of importance is cyclic variation in thresholds to pain stimulations linked to the menstrual cycle [32]. We did not include this variable in the present study.

### Effects of vibratory stimulation

The finding of this study that vibration may increase the threshold for experimentally induced pain is in accordance with earlier findings [10,15,16]. It is unclear how afferent signals elicited by vibration interfere with central transmission of nociceptive stimuli even though there are indications of mechanisms that include purinergic mechanisms [33,34], lowering of spinal substance P [35], but not, however, ligands to naloxone sensitive μ-opioid receptors [36,37].

Clinically, the effect of vibration stimulation in patients with different pain states varies between studies. Some demonstrated a pain lowering effect [1,3,10,30] whereas others were unable to show a statistically significant effect [38,39].

### Gender differences

The reason why women tend to have lower thresholds to some types of sensory stimulation including painful stimuli is obscure. The sensitivity to vibration as such does not appear to be different between men and women [40], indicating that differences in the intensity of the afferent signal was not the cause.

Another possibility is gender related differences in temporal summation. Temporal summation of painful stimuli has previously been shown to be larger in women [41,42], see however [43]. Whether this is also true for non-painful stimulation does not seem to be known. It could be that longer vibration duration in men would have cancelled out the observed threshold differences between men and women.

Even though gender related differences in pain perception have been reported [24,27], the gender differences appear to be rather small. The differences have been attributed to experimental, social, psychological and physiological factors, including the experimental setup and mode of stimulation, attention, emotional reactions including anxiety, willingness to report pain and gender of experiment assistant, and to catstrophizing [13,20,23,44]. Some of the complexity of the issue is also illustrated by the finding that repetitive mechanical painful stimuli were rated equal for the first stimulus, but higher in women than in men for the fifth and tenth stimulus, respectively [41], indicating central mechanisms. Also in line with this, less habituation in women than in men was found after intramuscular glutamate injections [26], and greater temporal summation following repetitive noxious stimulation [42].

## Conclusion

The main results of the present study are firstly that women have lower baseline DTs and PTs to electrical stimulation than men, and secondly that women, but not men, respond with an increase of the DTs and PTs immediately after vibration stimulation. Even though vibration had a statistically significant effect in women, further studies are needed to investigate the effect in a clinical context.

## Competing interests

The author(s) declare that they have no competing interests.

## Authors' contributions

LD supervised the sensory measurements in participating subjects, collected data and drafted the article. IL performed the statistical analysis, contributed to the writing of the results section, and produced tables and figures. TL conceived of the study, participated in its design and helped to draft the manuscript. CM helped to draft the manuscript, participated in its coordination and final design. All authors read and approved the final manuscript.

## Pre-publication history

The pre-publication history for this paper can be accessed here:


